# The Validation of the Chinese (Cantonese) Version of the Patient Dignity Inventory in a Hong Kong Palliative Care Setting

**DOI:** 10.1089/pmr.2023.0013

**Published:** 2023-08-30

**Authors:** Deepa Natarajan, Raymond Lo See Kit, Eric Liang Ka Shing, Alice Mok Ka Wai, Kevin Li Chi To, Harvey Max Chochinov

**Affiliations:** ^1^ Palliative Care Unit, Shatin Hospital, Kowloon, Hong Kong.; ^2^ Department of Psychiatry, University of Manitoba, Manitoba, Winnipeg, Canada.

**Keywords:** dignity, Hong Kong, palliative, Patient Dignity Inventory, validation

## Abstract

**Context:**

To assess and address a patient's dignity and dignity-related distress would greatly benefit patients who have advanced stage disease. The Patient Dignity Inventory (PDI) allows clinicians to identify sources of dignity-related distress for patients. The PDI should be evaluated for use in a local Chinese setting.

**Objectives:**

To validate the Patient Dignity Inventory Hong Kong-Chinese (Cantonese) version (PDI-HK) and assess the psychometric properties in patients in an inpatient palliative setting in Hong Kong.

**Method:**

The English version of the PDI was translated and back translated, then reviewed by a panel including a clinician, clinical psychologist, and nurse clinician. Recruited patients would complete the PDI-HK, the Chinese version of Hospital Anxiety and Depression Scale (HADS), the McGill Quality of Life Questionnaire-Hong Kong (MQOL-HK), and the Edmonton Symptom Assessment Scale. Psychometric properties including internal consistency, concurrent validity, test–retest reliability, and factor analysis were tested.

**Results:**

A total of 97 consecutive patients were recruited into the study. The mean PDI score was 51.85 (range 25–102). Cronbach's alpha was 0.953 (*p* < 0.001). Concurrent validity with the HADS and MQOL-HK questionnaire was established. Factor analysis showed four factors, namely Existential Distress, Physical Change and Function, Psychological Distress, and Support. These were similar to previous PDI validation studies.

**Conclusion:**

The PDI was translated into Chinese (Cantonese) and applied in an inpatient palliative care unit in Hong Kong, with adequate validity. The PDI-HK version can be further used in a larger Chinese population to assess and address dignity-related issues.

## Key Message

This is a validation study of the Patient Dignity Inventory (PDI) in a Hong Kong palliative care unit. The PDI was first published in English by Chochinov and his team. We have translated it into Chinese (Cantonese) and then validated it, with the results showing a good internal consistency. The notion of dignity in palliative care also applies in Chinese culture. With the PDI validated in Chinese, it can be used in the Hong Kong palliative care population, as well as any Chinese-speaking population in the world.

## Introduction

Dignity stems from two Latin words, *Dignitus* and *Dignus*, meaning *Merit* and *Worth*, respectively.^1^

Dignity can be defined as a person's self-worth or personhood, emphasizing the individuals past, their secrets, successes, lifelong work, together with their current strengths and interests, talents, vitality, and their context within a family and community. Hence, the need to have respect and regard for all that this individual represents—their dignity as a human person.^2^

Loss of dignity has been stated as one of the factors which leads to a loss of will to live, in addition to hopelessness, and burden to others.^2^ We cannot assume what a dignified death would mean for our patients, and it is important as clinicians that we understand the dignity of our patients, so as to structure the concrete, everyday care tasks, relationships, and decision-making processes for dying people.^3^

In 2002, Chochinov et al. published their results from a study on how dying patients in palliative care perceived their sense of dignity. It was found that patients who identified loss of dignity as being a great concern were far more likely that others to report psychological distress and symptom distress, heightened dependency needs, and loss of will to live.^3^

According to Chochinov's empirical model of dignity, three different themes of dignity have been identified, including Illness-Related Concerns, Social Dignity Inventory, and Dignity Conserving Repertoire.^4^ While a patient may have illness-related and social issues that may lead to the loss of dignity, through the Dignity Conserving Repertoire, dignity can be maintained.^4^

The Patient Dignity Inventory (PDI) thus developed by Chochinov is meant to be a measure of dignity-related distress and act as a screening tool to assess the different issues that have been reported to influence the sense of dignity. It can thus help clinicians identify a range of issues that can cause distress among patients. It had five factors that were labeled as Symptom Distress, Existential Distress, Dependency, Peace of Mind, and Social Support.^5^

The PDI has so far been translated into Spanish,^6^ Italian,^7^ German,^8^ Mandarin^9^ among other languages.

In Hong Kong, Cantonese is used as the main language and is most commonly spoken in the province of Guangdong and Macau. As there has been migration from this part of China to Canada, the United Kingdom, the United States, and Australia, there are large Cantonese-speaking populations in these countries.^10^ Cantonese is also quite distinct from Mandarin, in terms of the spoken and written language, and one may not understand Mandarin if they were Cantonese speaking and vice versa.

Previous research on dignity has shown that it is an important concept in the Chinese palliative population.^11–13^ Important aspects of dignity included autonomy, maintaining independence, preservation of social relationships, and filial piety.^11^

In particular, Ho et al. in 2013^14^ examined the generalizability of the Dignity model to older Chinese palliative patients in Hong Kong, and overall, the themes of Dignity in Chinese fit into the Model of Dignity by Chochinov, although one must keep in mind that the family unit is important in Chinese populations. In addition, some of the themes in Chochinov's model such as generativity/legacy may translate in a different manner in the Chinese population.

It would be useful to have a Cantonese version of the PDI so as to be able to assess dignity in this large population.

The objective of this study was to evaluate the psychometric properties of the Cantonese version of the PDI in an inpatient sample of palliative care patients. We determined factor structure characteristics, internal consistency, and concurrent validity through correlations with validated measures of anxiety and depression, and quality of life.

## Methods

This study was carried out in the palliative care unit of Shatin Hospital in Hong Kong. Approval from the Chinese University of Hong Kong—New Territories East Cluster Clinical Research Ethics Committee was obtained for the study.

### Translation procedure

The PDI was first translated by two independent bilingual translators, then back translated by one bilingual speaker, then further discussed and translations adjusted by the palliative team consultant physician, nurse consultant, and clinical psychologist, until a satisfactory translation was achieved and the Cantonese version completely fit with the English version.

### Participants

Consecutive patients who were admitted to the palliative care wards of Shatin Hospital during the period starting June 2018–March 2019 were assessed for eligibility of recruitment. Written informed consent was required to take part in this study.

Inclusion criteria included inpatients in Shatin Hospital with incurable malignancy, above 18 years old, able to give consent and complete a questionnaire in the Chinese language.

Exclusion criteria included cognitive impairment (assessed by Abbreviated Mental Test <6/10 as screening on admission), patients who are too ill to complete a questionnaire and patients with acute psychotic symptoms, such that they cannot complete the questionnaires.

### Data collection

Clinical correlations with dignity-related distress, including demographics such as age, sex, marital status, educational level, and religion, stage and type of malignancy, as well as time since diagnosis was documented, and functional status assessed using the Palliative Performance Score (PPS).^15^ Correlation with symptom assessment was done through the Chinese version of the Revised Edmonton Symptom Assessment System (ESAS).^16^

Patients completed the translated Patient Dignity Inventory—Chinese (PDI-HK), the McGill Quality of Life Questionnaire-Hong Kong (MQOL-HK), and the Hospital Anxiety and Depression Scale (HADS). The PDI has 25 questions, with each item rated on a 5-point Likert scale (1. Not a problem, 2. A slight problem, 3. A problem, 4. A major problem, and 5. An overwhelming problem). The PDI score can range from 25 to 125.

The McGill Quality of Life questionnaire is a multidimensional questionnaire developed for palliative care patients. It has been translated into MQOL-HK by Lo et al.^17^ Five domains of physical, psychological, existential, support, and sexuality are assessed.

The HADS^18^ is a self-rating instrument that measures the anxiety and depression levels of patients. The Cantonese-Chinese version of the HADS^19^ has been validated in the local population.

### Psychometric analysis

Descriptive statistical analysis was performed for demographic and clinical variables, with paired and unpaired *t*-test for continuous data and chi-square for categorical data. Internal consistency was evaluated using the Cronbach's alpha coefficient. Test–retest reliability was evaluated by giving a small sample of patients the questionnaires twice within a short period of time (48 hours) and calculated using the test–retest reliability coefficient.

Concurrent validity was assessed between the PDI-HK/ESAS, PDI-HK/HADS, and PDI-HK/MQOL-HK, measured by Spearman's rank correlation coefficient.

Factor analysis was also performed using Exploratory Factor Analysis, to confirm if this Chinese version of the PDI shows a similar factor structure to other validation studies in different languages. The factor analysis was done with principal axis factoring extraction method and orthogonal matrix. The selection of the factors for rotation was based on the eigenvalues >1 and analysis of the scree plot. The Kaiser–Meyer–Olkin (KMO) was used to evaluate the sampling adequacy, and the Bartlett's test of sphericity to test the study data derived from normal distribution with zero covariances.^20^

All statistical analysis was conducted using SPSS Version 1.0.0-2437. Results with *p*-values <0.05 were considered significant.

## Results

All patients who were admitted during that period were approached for participating in this study. After excluding those who were too ill, cognitively impaired, or not fit in any way to complete a questionnaire, a total of 97 patients completed the study.

Demographic and clinical characteristics of the participants are shown in Table 1. The mean age was 64.9, with 48.5% of the patients being female. The most prevalent diagnoses included breast and lung cancer, with most patients being diagnosed within one year. The PPS of patients ranged from 50 to 70. The mean score of the PDI was 51.85, standard deviation 19.73 (range from 25 to 110).

**Table 1.** Patient Demographics

Characteristics	***n***	%
Gender		
Male	50	51.5
Female	47	48.5
Marital status		
Single	13	13.4
Married	60	61.9
Divorced	12	12.4
Widowed	11	11.3
Unknown	1	1.0
Education		
None/kindergarten	10	10.3
Primary	40	41.2
Secondary or higher	39	40.2
Not available	8	8.3
Employment		
Retired	47	48.5
Working	25	25.8
Stopped work after diagnosis	24	24.7
Unknown	1	1
Religious beliefs		
Buddhist	13	13.4
Christian	9	9.2
Catholic	3	3.1
Traditional	1	1.1
Nil	68	70.1
Unknown	3	3.1
Primary (tumor)		
GI tract	14	14.4
Breast	15	15.5
Gynecological	8	8.2
Head and neck	3	3.0
Prostate	3	3.0
Lung	28	28.8
Hematological	9	9.3
Others	17	17.5
Time from diagnosis		
<1 year	44	45.4
1–5 years	35	36.1
>5 years	15	15.5
Unknown	3	3.1
PPS		
50	28	28.9
60	61	62.9
70	5	5.2
N/A	3	3.1

GI; N/A; PPS, Palliative Performance Score.

### Psychometric properties

Internal consistency for the 97 patients obtained a Cronbach's alpha of 0.953. For reviewing the Cronbach's alpha, by looking at the values by eliminating one item each time, there was no need to drop any items. Test–retest reliability was found to be good (baseline with 48-hour measurement), with intraclass correlation co-efficient being 0.915 (*p* = 0.000).

There was positive correlation of the PDI-HK total scores and the anxiety and depression scores of the HADS, which were statistically significant. There was stronger correlation with Depression scores of the HADS (*r* = 0.558, *p* = 0.000), and fair correlation with Anxiety scores (*r* = 0.453, *p* = 0.000) (Table 2).

**Table 2.** Correlation of PDI-HK and HADS

Correlations				
		PDI-total	Anxiety score	Depression score
PDI-total	Pearson correlation	1	0.453^**^	0.558^**^
	Significant (two-tailed)		0.000	0.000
	*n*	97	97	97

^**^ Correlation is significant at the 0.01 level (two-tailed).

PDI, Patient Dignity Inventory.

There were also statistically significant correlations between the PDI total scores and the McGill Quality of Life scores in the psychological and existential domain, with a strongest correlation (inverse correlation) with the existential subscale (*r* = −0.627, *p* = 0.000). This indicated the higher the PDI score was, the lower was the existential subscale score on the McGill questionnaire, and vice versa. There was also a moderate inverse correlation between the Physical subscale score and the PDI-HK (total) score, with *r* = 0.457, *p* = 0.001 (Table 3).

**Table 3.** Correlation of PDI-HK and McGill Quality of Life Scores

Correlations				
		PDI-total	Physical subscale	Existential subscale
PDI-total	Pearson correlation	1	−0.457^**^	−0.627^**^
	Significant (two-tailed)		0.001	0.000
	*n*	97	97	97

^**^ Correlation is significant at the 0.01 level (two-tailed).

Regarding correlation between the PDI-HK (total) and the ESAS (total), the correlation was weaker at 0.386 (*p* = 0.000).

The time it took to complete the questionnaire ranged between 10 and 45 minutes. Most patients completed the questionnaire with no difficulty. A majority of the patients did the PDI-HK with the assistance of someone to read out the questions; however, this also gave the opportunity for the patient to express their feelings in more detail. Eight patients completed the questionnaire on their own, without any assistance.

### Factor structure of PDI-HK

**FIG. 1.**
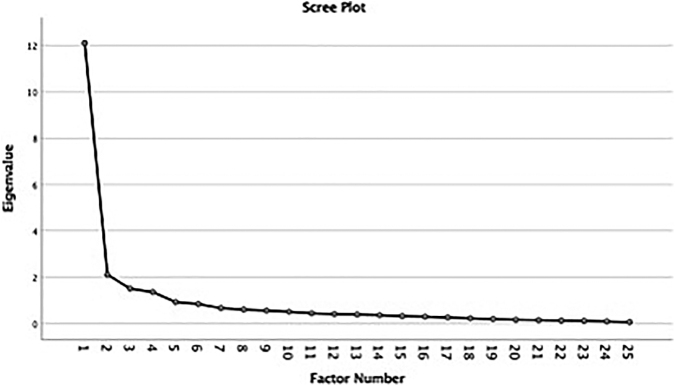
Scree plot of the eigenvalues plotted against the factor numbers supporting the four-factor choice.

The overall KMO value was 0.907. Bartlett's test of sphericity was X^2^ (300) = 1808.86 (*p* < 0.001), indicating the correlations between items were significant for principal axis factoring. According to the Eigenvalues >1, four factors were retained, which accounted for 68.319% of the data variance (Table 4). The examination of the scree plot of the eigenvalues plotted against the factor numbers also supported the four-factor choice (Fig. 1).

**Table 4.** Factor Matrix

	Rotated factor matrix^a^			
	Factor			
	1	2	3	4
P01		0.767		
P02		0.825		
P03		0.416		
P04		0.496		
P05			0.826	
P06			0.805	
P07	0.480	0.425	0.448	
P08	0.529			
P09	0.417		0.455	
P10		0.746		
P11		0.611		
P12	0.654			
P13	0.703			
P14	0.774			
P15	0.737			
P16	0.594			
P17	0.681			
P18	0.678	0.437		
P19	0.580	0.487		
P20	0.499			
P21				0.852
P22				0.737
P23	0.758			
P24	0.568			
P25	0.468			0.437

Extraction method: principal axis factoring. Rotation method: Varimax with Kaiser normalization.

^a^ Rotation converged in six iterations.

The 25 items of the PDI were hence allocated to one of the four factors identified (Table 5). The factors were labelled as Existential Distress, Physical Change and Function, Psychological Distress, and Support.

**Table 5.** Factors Obtained and Cronbach's Alpha Values

No.	Items	Cronbach's alpha
**First factor**	**Existential distress**	**0.949**
14	Feeling that life no longer has meaning or purpose.	
23	Feeling like I am no longer able to mentally cope with challenges to my health.	
15	Feeling that I have not made a meaningful and/or lasting contribution in my life.	
13	Not being able to carry out important roles (e.g., spouse, parent).	
17	Concern that my spiritual life is not meaningful.	
18	Feeling that I am a burden to others.	
12	Not feeling worthwhile or valued.	
16	Feeling I have “unfinished business” (e.g., things that I have yet to say or do; things that feel incomplete).	
19	Feeling that I don't have control over my life.	
24	Not being able to accept the way things are.	
8	Worrying about my future.	
20	Feeling that care needs have reduced my privacy.	
7	Feeling uncertain about my health.	
25	Not being treated with respect or understanding by others.	
9	Not being able to think clearly.	
**Second factor**	**Physical change and function**	**0.905**
2	Not being able to attend to bodily functions independently (e.g., needing assistance with toileting-related activities).	
10	Not being able to continue with my usual routines.	
1	Not being able to carry out tasks associated with daily living (e.g., washing, getting dressed).	
11	Feeling like I am no longer who I was.	
4	Feeling that how I look to others has changed significantly.	
19	Feeling that I don't have control over my life.	
18	Feeling that I am a burden to others.	
7	Feeling uncertain about my health.	
3	Experiencing physically distressing symptoms (such as pain, shortness of breath, nausea).	
**Third factor**	**Psychological distress**	**0.855**
5	Feeling depressed.	
6	Feeling anxious.	
9	Not being able to think clearly.	
7	Feeling uncertain about my health.	
**Fourth factor**	**Support**	**0.733**
21	Not feeling supported by my community of friends and family.	
22	Not feeling supported by my health care providers.	
25	Not being treated with respect or understanding by others.	

## Discussion

This study provides the first Cantonese version of the PDI. This instrument was originally designed and validated in English by Chochinov et al., and later translated and validated into Spanish,^6^ Italian,^7^ German,^8^ and Mandarin,^9^ and other languages with time. To have a Cantonese version makes it possible to be used in any country with a Cantonese-speaking population, which includes many countries all over the world.

The patients in this study were patients with incurable cancers, as was the initial PDI study. It was limited to only cancer patients, so as to keep the study population more controlled. Our patient group was all inpatient; hence, the environment was also standardized. We did not perform the PDI-HK on outpatients, as was done in the Spanish validation study,^7^ but this would be worthwhile to look into as there was a difference between the two settings noted in that study. Most patients were diagnosed within one year of performing the PDI. This period ranged between 6 and 24 months in other studies.

Cronbach's alpha coefficient was high, which is similar to previous validation studies (0.89–0.96).^6–9^ A reliability coefficient is considered acceptable above 0.7.

Test–retest reliability (baseline with 48-hour measurement) was tested using the intraclass correlation co-efficient, with a result of 0.915 (*p* = 0.000), which is excellent.

The test–retest interval in the original PDI study was 24 hours, which is similar to our study. A time interval that is not too long would be appropriate, as patient conditions may change, especially in an inpatient setting.

In Chochinov's initial study on the PDI,^5^ there were five factors in their analyses, including Symptom Distress (3, 5, 6, 7, 8, 9), Existential Distress (4, 11, 12, 13, 14, 18), Dependency (1,2, 20), Peace of Mind (15,16,17), and Social Support (21,22,25).

There were four factors obtained in this present study, with all four factors having an acceptable Cronbach's alpha. Factor one included items related to not being able to have a meaningful life, or making a lasting contribution, or life not having meaning or purpose, not being able to carry out roles, not having control over one's life. None of these have much relation to ones' physical function or symptoms, and yet they have such a large impact on ones' dignity. This would be similar to Chochinov's factor of Existential Distress; hence, we named it Existential Distress. Although in the original study, there were only six items, whereas in our study, more factors fit into this category.

Factor 2 included six items which related more to physical function or loss of physical independence. We hence named it as Physical Change and Function. We did see many patients who were very concerned about their change in function from what they used to be able to do and expressed their hopes to be able to return to a function where they do not have to depend on others.

Factor 3 included questions on depression, anxiety, and not being able to think clearly. Hence, we named it as Psychological Distress. Factor 4 included being supported by others whether it be family or health care professionals; or being understood by others, hence, was named Support.

This factor structure accounted for a relatively high percentage of data variance (68.319%), with previous studies having similar percentages (58%–71%). In the previous PDI validation studies, factors identified ranged between three to five, but overall were similar to the original study.

In our study, the PDI correlated with the ESAS and HADS. It also correlated with the McGill QOL questionnaire, which also has similar dimensions such as Physical, Psychological, Existential, and Support.

In particular, there was stronger correlation with the existential scale of the McGill questionnaire rather than the physical scale, as well as strong correlations with the depression part of the HADS. In contrast, there was lower correlation of the PDI with the ESAS, which is mainly physical symptoms. This indicated that the PDI, while it had some element of physical distress, overall had more emphasis in other aspects, such as existential or psychological aspects.

Patients were able to complete the questionnaire with ease, in terms of understanding the questions or the time it took to complete it. Only a small number of patients completed the questionnaire by themselves. Most completed it with the aid of one of the researchers, and this provided an opportunity for the researcher to explore and the patients to talk about what was important for them. This conversation became very fruitful and resulted in a closer relationship between the doctor and patient. PDI can be used as an intervention on its own, with possible therapeutic value, as seen in the papers by Rullan^21^ and Chochinov,^22^ which studied what the process of performing the PDI meant to clinicians or other members of the team. What they found that instead of doing the PDI as a self-filled questionnaire, by doing the PDI with the patient led to a profound conversation on the meaning of life, dignity, and other sensitive, key issues related to the process of the illness, hence suggesting that the PDI had intrinsic therapeutic value.

## Conclusion

The PDI was translated into Chinese (Cantonese) and applied in an inpatient palliative care unit in Hong Kong, with adequate validity. It was also significantly correlated with other measures of depression and psychological and existential subscales of the McGill Quality of Life questionnaire.

The PDI was also found to be a useful tool to discuss issues important to patients, and it was accepted well by our group of patients.

The PDI-HK (Cantonese) version can be further used in a larger Chinese population all over the world to assess dignity-related issues.

## Limitations

One of the limitations of the study was that it was done in a single hospital setting. However, this can now be used in larger and multiple settings in future research. The sample size was also not very large. While there is no absolute rules for the sample size used to validate a questionnaire, given the variations in the types of questionnaires, sometimes a range of 5:1 up to 30:1 are seen.^23^ Overall, a bigger sample size would strengthen the study.

Also, test–retest analysis was done only on a small sample, and it would be better to do it on a larger sample.

## Abbreviations Used

AMT: Abbreviated Mental Test
ESAS: Edmonton Symptom Assessment System
GI: gastrointestinal
HADS: Hospital Anxiety and Depression Scale
KMO: Kaiser–Meyer–Olkin
MQOL-HK: McGill Quality of Life Questionnaire-Hong Kong
N/A: not available
PDI: Patient Dignity Inventory
PDI-HK: Patient Dignity Inventory—Chinese
PPS: Palliative Performance Score

## References

[1] Kennedy G. The importance of patient dignity in care at the end of life. Ulster Med J 2016;85(1):45–48. 27158166 PMC4847835

[2] Street AF, Kissane, DW. Constructions of dignity in end-of-life care. J Palliat Care 2001;17(2):93–101. 11477991

[3] Chochinov HM, Hack T, Hassard T, et al. Dignity in the terminally ill: A cross-sectional, cohort study. Lancet 2002;360(9350):2026–2030; doi: 10.1016/S0140-6736(02)12022-8 12504398

[4] Chochinov HM, Hack T, McClement S, et al. Dignity in the terminally ill: A developing empirical model. Soc Sci Med 2002;54(3):433–443; doi: 10.1016/s0277-9536(01)00084-3 11824919

[5] Chochinov HM, Hassard T, McClement S, et al. The Patient Dignity Inventory: A novel way of measuring dignity-related distress in palliative care. J Pain Symptom Manage 2008;36(6):559–571; doi: 10.1016/j.jpainsymman.2007.12.018 18579340

[6] Rullan M, Carjaval A, Nunez-Cordoba JM, et al. Spanish version of the patient dignity inventory. Translation and validation in patients with advanced cancer. J Pain Symptom Manage 2015;50(6):874–881; doi: 10.1016/j.jpainsymman.2015.07.016 26342725

[7] Ripamonti Cl, Buonaccorso L, Maruelli A, et al. Patient Dignity Inventory (PDI) questionnaire: The validation study in italian patients with solid and hematological cancers on active oncological treatments. Tumori 2012;98(4):491–500; doi: 10.1177/030089161209800415 23052167

[8] Sautier LP, Vehling S, Mehnert A. Assessment of patients' dignity in cancer care: Preliminary psychometrics of the German version of the Patient Dignity Inventory (PDI-G). J Pain Symptom Manage 2014;47(1):181–188; doi: 10.1016/j.jpainsymman.2013.02.023 23830532

[9] Li YC, Wang HH, Ho CH. Validity and reliability of the Mandarin version of Patient Dignity Inventory (PDI-MV) in cancer patients. PLoS One 2018;13(9); doi: 10.1371/journal.pone.0203111 PMC6126806 30188928

[10] https://worldmapper.org/maps/cantonese-language-2005/ [Last accessed: July 16, 2023].

[11] Sham M. Human dignity in patients with terminal illness. 2009. Available from: https://www.yumpu.com/en/document/view/51402824/full-paper-human-dignity-in-patients-with-terminal-illness [Last acceessed: July 16, 2023].

[12] Zhai X, Qiu RZ. Perceptions of long-term care, autonomy, and dignity, by residents, family and caregivers: The Beijing experience. J Med Philos 2007;32:425–445; doi: 10.1080/03605310701631695 17924270

[13] Chan HM, Pang S. Long-term care: Dignity, autonomy, family integrity, and social sustainability the Hong Kong experience. J Med Philos 2007;32:401–423; doi: 10.1080/03605310701631661 17924269

[14] Ho AH, Chan CL, Leung PP, et al. Living and dying with dignity in Chinese society: Perspectives of older palliative care patients in Hong Kong. Age Ageing 2013;42(4):455–461; doi: 10.1093/ageing/aft003 23443510

[15] Anderson F, Downing GM, Hill J. Palliative Performance Scale (PPS): A new tool. J Palliat Care 1996;12(2):5–11. 8857241

[16] Bruera E, Kuehn N, Miller MJ, et al. The Edmonton Symptom Assessment System (ESAS): A simple method for the assessment of palliative care patients. J Palliat Care 1991;7:6–9; doi: 10.1177/082585979100700202 1714502

[17] Lo RSK, Woo J, Zhoc KCH, et al. Cross-cultural validation of the McGill Quality of Life questionnaire in Hong Kong Chinese. Palliat Med 2001;15:387–397; doi: 10.1191/026921601680419438 11591090

[18] Zigmond AS, Snaith RP. The Hospital Anxiety and Depression Scale. Acta Psych Scand 1983;67:361–370; doi: 10.1111/j.1600-0447.1983.tb09716.x 6880820

[19] Leung CM, Wing YK, Kwong YK, et al. Validation of the Chinese-Cantonese version of the Hospital Anxiety and Depression Scale and comparison of the Hamilton Rating Scale of Depression. Acta Psych Scand 1999;100:456–461; doi: 10.1111/j.1600-0447.1999.tb10897.x 10626925

[20] Kaiser HF. The application of electronic computers to factor analysis. Educ Psychol Measure 1960;20(1):141–151; doi: 10.1177/001316446002000116

[21] Rullan M, Arantzamendi M, Carvajal A, et al. The Patient Dignity Inventory: Just another evaluation tool? Experiences with advanced cancer patients. Palliat Support Care 2018;16(1):73–79; doi: 10.1017/S1478951517000517 28635584

[22] Chochinov HM, McClement S, Hack T, et al. The Patient Dignity Inventory: Applications in the oncology setting. J Palliat Med 2012;15(9):998–1005; doi: 10.1089/jpm.2012.0066 22946576

[23] Tsang S, Royse CF, Terkawi AS. Guidelines for developing, translating, and validating a questionnaire in perioperative and pain medicine. Saudi J Anesth 2017;11(5):80–89; doi: 10.4103/sja.SJA_203_17 PMC5463570 28616007

